# Voluntary Assisted Dying/Euthanasia: Will This Have an Impact on Cancer Care in Future Years?

**DOI:** 10.1007/s11864-023-01126-8

**Published:** 2023-08-03

**Authors:** Jennifer Philip, Brian Le, Camille La Brooy, Ian Olver, Ian Kerridge, Paul Komesaroff

**Affiliations:** 1 Department of Medicine, University of Melbourne, Victoria Pde, Fitzroy 3065, Melbourne, Victoria Australia; 2 Palliative Care Service, St Vincent’s Hospital, Melbourne, Victoria Australia; 3 Parkville Integrated Palliative Care Service, Peter MacCallum Cancer Centre & Royal Melbourne Hospital, Melbourne, Victoria Australia; 4 Public Health & Preventive Medicine, Monash University, Monash, Victoria Australia; 5 University of Notre Dame of Australia, Sydney, NSW Australia; 6 Haematology Department, Royal North Shore Hospital, St Leonards, NSW Australia; 7 Sydney Health Ethics, University of Sydney, Sydney, NSW Australia; 8 Department of Philosophy, Macquarie University, Macquarie, NSW Australia; 9 Monash University, Monash, Vic Australia

**Keywords:** Euthanasia, Voluntary assisted dying, Medically hastened death, Palliative care, Cancer

## Abstract

In considering the impact of medically hastened death (MHD) on cancer care, a wide range of variables needs to be considered including demographic factors, diagnoses, local cultural factors, and the legislative frameworks in place. Here, we present a synthesis of recently available published literature and empirical data collected following legislative change to enable MHD in Victoria, Australia to explore in detail the potential impact of MHD on cancer care with a focus on patients/families and professional groups. Our findings reveal that for patients and families, both physical and existential distress frequently underlie MHD requests, with the latter less readily recognised by health professionals. The responses of those around the patient making the request may have a very significant impact on relationships within families and upon the nature of the subsequent bereavement. For palliative care, while differing views may remain, it appears that there has been some accommodation of MHD into or alongside practice over time. The recognition of a shared commitment to relief of suffering of palliative care and MHD appears a helpful means of establishing how these practices may co-exist. In cancer practice more broadly, as individual professionals reflect upon their own roles, new relationships and pathways of patient movement (or referral) must be established in response to patients’ requests. Our findings also highlight many unanswered questions in understanding the impact of MHD, including that upon those dying who choose not to access MHD, First Nations peoples, the participating health professionals’ longer term, and the relief of suffering itself. A systematic approach to the evaluation of MHD legislation must be adopted in order to understand its full impact. Only then could it be determined if the aspirations for such legislative change were being met.

## Introduction

Internationally, the landscape of medically hastened death (MHD), euthanasia, and physician-assisted suicide, often also referred to as “voluntary assisted dying” (VAD) has changed in recent years in a way that raises questions about the future treatment of cancer and potentially other terminal illnesses.

Clinician-administered and self-administered MHD have been legal in the Netherlands and Belgium since 2002, while self-administered MHD has been permitted in Switzerland since 1918, and in the American state of Oregon since 1997. There have been shifts over time around the ability to access these laws; for example, in 2014 Belgium extended the law to allow terminally ill children and people with psychiatric disorders access to MHD [1, 2]. Where available, the number of people accessing MHD is increasing; however, until recently, the numbers of jurisdictions to which these laws have applied have been relatively stable [3, 4]. In the past 8 years, the total number of jurisdictions with overarching laws enabling some form of MHD has increased substantially and now also includes Canada, Spain, all states in Australia, Germany, Colombia, New Zealand, and many states within the USA (https://end-of-life.qut.edu.au/assisteddying). The evolution of legislative instruments in this field is in varying states of maturity, as a result of which there remains a need for the details of these laws, and ways in which they may be improved, to be carefully assessed.

The authors of this paper are situated in Australian jurisdictions where the implementation of voluntary assisted dying laws is recent (since 2019) or, in some states, still being awaited. In this country, as in many other parts of the world, public and professional debates about MHD centred around a small number of areas of contention, including: the relief of suffering and especially unrelieved pain; the value of “life” and of “autonomy”; independence and dependence; the nature of suicide; quality of and access to palliative care; the impact on persons (patients and family members) and clinicians; the impact upon the practice of medicine; and the impact upon society. While these debates have been prosecuted, often with vehemence, the systems in place to capture data that may inform (but not settle) them have been comparatively limited.

Formal legislative requirements for monitoring the impact of MHD laws in most instances record limited details—as in Victoria, Australia, where the official data report no more than age, sex, broad geographical location, country of birth and language, level of education, living situation, access to palliative care, and broad diagnosis [5]. In addition, “process” information is largely limited to a record of the time taken to access VAD from the first request. Information about motivation, symptom burden, family circumstances, financial and treatment costs, gaps in care, and decision-making processes is not available, even in rudimentary form. Indeed, in Australia as in other jurisdictions internationally, the most detailed data on MHD are the result of competitively funded research grants that are time-limited and not “built in” to systems of legislation or monitoring (personal communication, workshop [6]).

## Cancer diagnosis most common

In Victoria, where the system of VAD came into effect in 2019, people accessing it were male (54%), older (median age 73 years), spoke English at home (93%) and had a cancer diagnosis (82%) [5]. Similarly, in Canada, people accessing MHD were more likely to have a cancer diagnosis (64.4% vs. 27.6% general Ontario decedent cohort) [7] while cancer accounts for more than 70% of MHD in Belgium and The Netherlands [8] and the majority (79%) of those receiving prescriptions for self-administered MHD in Oregon, USA [9].

The predominance of cancer diagnoses invites analysis. It may be that this reflects common experiences of cancer illnesses or fears about cancer deaths [10]. Alternatively, it may be an effect of procedural and legislative structures. For example, in Victoria, the legislation limits eligibility to cases where a consulting physician assesses the person as having a 6 month or less prognosis [11]. People with chronic non-malignant diseases such as chronic obstructive pulmonary disease typically have an illness course characterised by acute exacerbations and stabilisation meaning accurate prognostication is difficult [12]. In addition, combinations of such diseases conferring multi-morbidity and, in many cases, frailty are common, further complicating prognostic predictions and reducing the likelihood of satisfying eligibility criteria to access MHD [13, 14].

Given that those with cancer diagnoses make up the majority of those accessing MHD, it is appropriate to focus upon this population when assessing the impact of MHD legislative frameworks.

In considering the impact of MHD on cancer care, a wide range of variables needs to be considered, including demographic factors, diagnoses, local cultural factors, and the legislative frameworks themselves. In addition, the specific effects on different population groups need to be considered, namely on patients and their families, professional and non-professional carers (including palliative care services) and on the wider community.

Here, we present a synthesis of recently available literature focusing on articles published in English since 2019, and supplement this with empirical data collated as part of a longitudinal research project to understand the impact of VAD legislation through interviews with patients (*n* = 36), family/community carers (*n* = 42), and health professionals (*n* = 67) in Victoria, Australia, where VAD legislation came into effect in June 2019.

We present data regarding the effects of VAD legislation around the impacts on patients and families, on palliative care and on cancer care and conclude by considering the outstanding unanswered questions, responses to which should help guide the future responses of the cancer community.

## Impact on patients and families

Fear of unrelieved pain remains central to public support for MHD [8, 15]. Prior to legislative change, the public debates frequently centred upon the existence of pain causing “intolerable suffering” which required urgent redress with MHD, with frequent claims about such an option constituting a mark of a “humane society” [16]. However, empirical research exploring the concerns and distress that prompt requests for MHD often concludes that pain is not the central motivation. Instead, for example, in Belgium, pain was prompting MHD requests in only about half the cases, with “loss of dignity” cited in 52% (Belgium) and 61% of cases in the Netherlands [8].

A Canadian study of 112 patients who accessed MHD suggested that while disease-related symptoms were given as the first or second most important reason for requesting assisted death by 67 people (59.8%), similar numbers (52.7%) cited “loss of autonomy”, 49% spoke of their loss of ability to enjoy activities, and 24% feared future suffering [9]. Variath et al. highlighted being a burden, financial difficulties, and an inability to integrate into the community as drivers of requests for MHD [17], with similar relational concerns common amongst patients interviewed in our study (see box, statement a).

In a qualitative review of the views of psychiatric patients who requested MHD, Verhoffstadt showed the complexity and, for some, the ambiguity behind requests. In that study, most patients described a state of feeling emotionally worn-out as a result of the many accumulated misfortunes, leading to the all-pervasive sense that life was no longer worth living. While some patients longed strongly for death, others expressed ambivalence towards the idea of death, and some even revealed that their request for MHD was made in part to hear that they would be ineligible, which might thereby restore some hope and meaning in their lives. The interviewed patients stated that they saw MHD as being more dignified and acceptable than suicide—both for themselves and for those close to them [19].

Conversely, in a study of mostly older people requesting MHD who were not severely ill, suffering (which was largely defined in physical domains) was commonly cited as driving the request [20]. Leboul highlighted the potential for positive opportunities that may arise following a request for MHD even when it is not legally available [21]. In inpatient palliative care patients, such a request was seen as a call to recognise intolerable suffering, to change clinical practice, to reclaim a sense of removal of medical controls and to imagine “a desirable future for oneself’”. As such, even in the absence of likely fulfilment of the wish, the request created an opportunity to navigate care differently—to discuss, consider, and negotiate therapeutics, to enhance a sense of mastery in circumstances previously considered without such opportunities [21].

The role of the family in MHD requests focuses attention on the relationships involving patients and families, families and clinicians, and patients, families and clinicians, with the possibility of enhanced communication and greater appreciation of the complexity of the issues. Variath et al. have noted that when family members were opposed to MHD, patients may make requests to clinicians without disclosing them to families. This can create issues of privacy and ultimately of consent to disclose if the patient does ultimately proceed down a pathway of accessing MHD. When physicians displayed empathy and a willingness to openly explore concerns and options, this encouraged positive interactions with and between patients and family members [17]. In the same study, when patients requested MHD for “relief of unbearable suffering”, physicians found it easier to concur when the source of suffering was visible—for example, where there was a physical symptom rather than an existential or social cause, such as the patient feeling a burden or being socially isolated. In the absence of visible suffering, family members too found it more difficult to support these requests [17].

Data from carers interviewed as part our project similarly reflected this idea (see box, statement b).

Instances were described by clinicians of patients making a request in advance for assisted dying but subsequently having reduced decision-making capacity and that family members had resisted these stated directions. Physicians in these cases noted the complexities in seeking to uphold the previously stated wishes of the no longer competent patient when families were advocating otherwise [17].

**Box** Empirical data from qualitative interviews with patients, family/community members and health professionals.

**Table:** 

Statements
(a) “I guess my fear really is that I just won’t be able to contribute anymore…I will actually genuinely be that word that everyone uses, being a burden. That’s my biggest fear.” (Patient) (b) “This comes to the core of it all, people need to see suffering… Suffering is physical pain. Now, dad wasn’t in physical pain… Therefore, that created a lot of unease for people.” (Family carer) (c) “This time last year… I would say it was almost kind of taboo and not allowed to be discussed in the ward. … this year it doesn’t seem like so much of an issue. It’s like, “Oh yeah, they want VAD, it’s their decision. Whatever.” (Palliative care Physician) (d) “When a patient is requesting MAID [Medical Assistance in Dying], most of the resources have been sucked up by that one case…And all of the high-quality palliative care that we do falls by the wayside for the other patients.”[18] (e) “Their request becomes the focus… and they are absolutely determined that nothing will impact their competency. So it ends up …(they are) entering the terminal phase, very symptomatic and refusing medications that would manage their suffering as they don’t want to be sedated or lose capacity” (Palliative care Physician) (f) “I think that voluntary assisted dying, with self-administration, you’re really not playing an active role there. … Whereas practitioner administration, you’re actually doing something and your actions are causing a death… Even though on an intellectual level I was totally comfortable with my actions, there was real sense that I was doing something…” (Oncologist)

The impact of MHD upon family bereavement outcomes has attracted attention. A systematic review identified eight studies exploring this issue and found that people bereaved by MHD had similar measures relating to disordered grief, mental health, and post-traumatic stress to those people who were bereaved by a natural death. Of note, the opportunity of being involved in decisions around end of life and having fulfilled the deceased persons’ wishes were identified as helpful to grief, while secrecy around the death had a negative effect [22]. Others highlighted the ability to say goodbye and the rituals enacted surrounding the MHD as positive experiences [23]. Qualitative studies of bereaved family members convey the sense of pressure felt to facilitate an “ideal death”, while balancing administrative processes and the need for ensuring capacity underlined all activities [24].

The responses of First Nations peoples to legislation passed in this area have revealed a similar range of views, with some expressing support and others believing it to be unnecessary and potentially damaging. Amongst First Nations people in Australia there are many additional factors including a history—past and current—of colonialism and dispossession, of institutional racism and of distrust of existing systems of government and health—all of which are incorporated into the complexity of formulating a response to MHD [25]. Furthermore, the approaches to decision-making in First Nations communities may be at odds with those taken by the MHD legislative processes. The latter almost invariably assume the existence of autonomous individuals who may, following deliberation of the possible benefits and harms, reach a conclusion about their own preferred course of action. By contrast, for many First Nations peoples, decisions may not be purely individual matters but may involve immediate family, elders, and broader community members [26, 27]. These approaches to decision making are likely to be particularly evident in decisions around MHD.

## Impact on palliative care

The debates about MHD prior to legislation being introduced frequently centred upon a false dichotomy of palliative care or MHD. Such debates often involved discussions that included statements that “if palliative care were available then MHD would be unnecessary”, “the solution to suffering is broader access to palliative care”, or conversely, that “palliative care was tried and failed” to relieve the suffering experienced [28]. Funding and resources for palliative care services have been a focus of a number of representations to legislative bodies in the setting of MHD public debates [29]. Of note, the public understanding of palliative care, including of its goals, philosophy and practices, is poor [30], while media representations of palliative care in times of MHD debate have been documented to be deficient, with MHD represented as enhancing choice and relieving suffering and palliative care as limiting choice and forcing prolongation of suffering, precisely because it does not intentionally hasten death [31]. Yet palliative care clinicians and many patients consider palliative care a creative option—providing relief of current and future suffering through symptom relief, access to information, counselling, and care for the whole person and their family [32]. To facilitate a more nuanced debate in those jurisdictions where future legislation is considered, it has been proposed that palliative care should actively seek to shape, rather than react to, the debate, with a particular aim of enhancing community understanding of its goals and activities [33].

One concern expressed has been that investment in MHD may have an impact upon investment in palliative care—either limiting growth or indeed effectively reducing funding. The impact of this, it has been suggested, would be to further increase the demand for MHD as people experience unaddressed suffering that could otherwise have been ameliorated in a well-serviced system [34]. The impact upon service development is, however, difficult to assess. In a study of European countries between 2005 and 2012, spanning both MHD permissive and non-permissive countries, there was, by 2012, comparative palliative care service activity across all countries, reflecting a substantial increase in palliative care, particularly in the Netherlands following MHD legislative introduction in 2002. Interestingly, these had begun at a lower level in the Netherlands, at 8.45 services per million inhabitants, later growing to 15.32 by 2012, compared with the UK, which only increased minimally from 14.73 to 15.43 [34]. It is not possible to state if funding to support MHD services has either detracted from or been accompanied by expansion of palliative care services in other jurisdictions.

Concerns that MHD may be chosen as an alternative to palliative care [35, 36] have not generally been born out. For example, 74% of people who access MHD in Canada, and over 80% in Victoria, have had palliative care input into their care [5, 7], while in Belgium, palliative care clinicians are involved in the decision-making for 60% of those who go on to have a MHD death [37].

Data from recent qualitative studies of the rapidly accumulating experience of MHD have largely supported these conclusions. For example, palliative care professionals have highlighted the tension in practice between honouring the patient’s “personal autonomy and control” at the end of life and honouring a commitment to the sense that life is “inherently valuable and meaningful” [38]. For these interviewees, the commitment to the “compassionate care for the dying person” was a unifying value and helpful in working in this space of co-existence [38].

The relationship of co-existence between the practices of palliative care and MHD across countries where MHD is legal, has been shown to cover a range of stances from supportive, integrated and cooperative through to ambivalent and then to opposed, and mutually exclusive [39]. In interviews with clinicians and policy makers from Quebec, Canada; Flanders (Belgium) and Oregon (USA) different themes were identified across the three jurisdictions [40]. In Quebec a set of contested relationships were evident in the context of a lack of knowledge about and access to palliative care. In Flanders an adoption of an integrated approach with palliative care and MHD developing contemporaneously was evident, although it was noted that patients at times viewed palliative care as a “barrier” to MHD, meaning it was not always offered or accepted, and others expressed concern about a progressive liberalisation of assisted dying laws. In Oregon there was some variation in the protocols enacted at the end of life creating fear of breaching policies or being the subject of litigation, along with concerns about access to medications that were not always available or affordable [40].

In Victoria, an accommodation of MHD by palliative care services appears to have developed, with a lessening of anxiety surrounding its practices and procedures (see box statement c).

This increasing tolerance amongst palliative care providers has also been described in Canada where after initial strong opposition, over time, a position developed of co-existence with MHD [18]. Within this state of co-existence, however, several key stress points for palliative care continue to be highlighted. First, the significant time taken to respond to and facilitate the wishes of a person seeking MHD has a workload impact on palliative care. These add extra tasks to usual palliative care delivery which must also be part of the person’s care. In Victoria, Australia senior clinicians have taken on the responsibility for MHD, thereby protecting their junior colleagues from these tasks. Even for those not involved in MHD, there are, in addition to discussions with the patients (and their families as permitted), multiple interactions with colleagues to enact referrals, assessments, and all the other the processes that unfold up to the point of MHD death. These tasks inevitably have an impact upon the delivery of services to other patients who are not seeking MHD who receive less senior clinician attention (see box d).

Secondly, and likely unforeseen by legislators, for some patients, high-quality end-of-life care is actually undermined by the possibility of MHD [18]. Clinicians have highlighted situations where patients, in the last weeks (or days) of life, unexpectedly express a strong desire to access MHD. In seeking to enable exploration of that pathway, such patients may refuse medications and interventions that would usually be part of standard, good-quality end-of-life care, apparently for fear of compromising their decision-making capacity during the requested MHD assessments. For many such patients, the proximity to natural death means they are not able to access MHD and simultaneously they are forsaking good relief of symptoms and suffering at the end of life. Tragically, they lose on both fronts (see box e).

Thirdly, and related to these, is the impact on palliative care practitioners themselves. Despite some accommodation over time with reduced anxiety amongst a number of palliative care practitioners, the emotional toll was described by many [18]. In addition to the distress felt by some when a patient undertakes MHD while under their care, additional organisational processes needed to be enacted—for example, a manager might have to assume ward nursing duties to care for a patient in order to relieve the rostered nurse who has a personal objection to MHD. All of these factors may have an impact upon the day-to-day tasks of providing palliative care and may be distressing for some. Of course, these negative experiences do not negate that many other clinicians have described a deep satisfaction from being part of a care team at a time of MHD enactment [41].

In summary, the impact of MHD upon palliative care providers suggests that there is an expansion of workload associated with this practice, even when the practitioners are not directly participating. There is also a concurrent emotional impact that can be heightened when the delivery of MHD or its exploration affects the delivery of what the treating clinical team considers to be the best palliative care.

## Impact on oncology and other specialities in medicine

The discussion of the impact of MHD on cancer care extends beyond palliative care to multiple other settings and disciplines. While cancer patients remain the largest group to seek and access MHD, the proportion of oncologists who participate in providing MHD is disproportionately smaller—just 16% of those participating physicians in Australia identified as oncologists [42], and there is surprisingly little literature detailing the impact of MHD on the field of cancer care. Thus, the effect upon other craft groups and upon medicine more broadly is the subject of the following discussion.

The culture of medicine and its prevailing views have an impact upon individual physicians and how they interact with their patients and the broader community. Medical students at the start of their training are more likely to support legislation enabling MHD than those in the final years of training (65% vs. 39%), suggesting exposure to medical education and culture may have an impact [43]. Meanwhile, in a multisite survey of 5690 hospital staff in Victoria, Australia, overall 73% of clinicians indicated support for MHD legislation, but substantially fewer numbers of medical specialists expressed willingness to participate (40%) and this willingness was significantly less amongst those doctors who specialised in aged care or palliative care (27%) [44].

A possible challenge for medical practitioners is the pressure on them to actively participate in the processes of patient access to MHD. In a study of Dutch general practitioners who make up the majority of administering physicians in that country, many felt pressure from patients to accede to requests including when there was uncertainty about eligibility and simultaneously counterpressure from relatives to resist such requests with significant time required to navigate these discussions [45]. Furthermore, participating medical practitioners may have a differential response to the different administration approaches of MHD, namely self-administered and physician administered MHD. While both are available in the Netherlands and in Canada, physician assisted MHD has been the overwhelming preference for patients accessing these laws [46, 47]. In Victoria, Australia, where self-administration is the available option unless the patient is unable to administer themselves, physicians have described their differential responses to participation (see box f).

## Unanswered questions

The above discussion suggests a series of conclusions that may be drawn about the impact of MHD upon cancer care. For patients and families, in addition to physical distress, concerns that may be considered social or existential are frequently cited as motivations for requests, and these are often less readily recognised by health professionals. The responses of those close to the patient making the request may have a very significant impact on relationships within families, and within health care, as well as an impact upon the nature of the subsequent bereavement. For palliative care, while differing views may remain, it appears that there has been some accommodation of MHD into, or alongside practice over time. The recognition of a shared commitment to relief of suffering of palliative care and MHD appears a helpful means of establishing how these practices may co-exist. While in oncology practice, a similar process of determining individual professional practices must take place as MHD is introduced. With most not participating in the process, new relationships and pathways of patient movement (or referral) must be established in response to patients and colleagues who choose to participate. Meanwhile, the extent of participation (or not) appears to be a matter for ongoing reflection for some oncologists.

These conclusions are subject to qualifications and limitations, largely due to the lack of adequate data in crucial areas such as, but not limited to, the impact of MHD legislation on the care of the dying who do not choose MHD, on First Nations peoples, the role of clinician communication, the relationships with and between health service providers and the longer-term impact upon participating health professionals.

The method by which the impact of legislative change to enable MHD should be evaluated remains a significant and unanswered question. The aspirations and concerns voiced when legislation was introduced can and should be systematically monitored and responses provided. To date, evaluation has centred on population-level accounts of the numbers and limited clinicodemographic information of people participating, results of surveys of targeted groups (clinicians, general public) and, largely single centre, qualitative studies, usually of small samples which seek to provide more in-depth examination of motivations for requests and the outcomes for patients, families, and clinicians.

Measures of success or otherwise are generally discussed in terms of “processes”, such as the time taken between the first request and death and the difficulty of collating evidence to satisfy eligibility criteria [48]. Other discussions about impacts have centred on technical aspects, such as the details of the wording in the laws, which in some cases have been said to constrain clinical care delivery—for example, by prohibiting clinicians from raising MHD, which has meant for some clinicians an avoidance of conversations for fear of prosecution [49].

It is not clear what drives the observed increase over time of the numbers of people accessing MHD. Nor is it clear if the evolution of eligibility criteria for VAD are in response to reflection and perceived deficiencies in existing legislation or another process of community change. Furthermore, the relief of suffering—the underlying premise for the need for MHD legislation and a common aspiration of all those who participate in the debates around legislation—is not readily evaluable in the broader community. These community and society level evaluations are currently not available.

Accordingly, at the present time, the impact of MHD legislation remains uncertain. There is, therefore, a need for the development of a comprehensive framework that both allows the impact of MHD legislation upon the community to be assessed over time, and the effects in different communities to be compared. Such a framework must address the extent to which legislation relieves suffering for people who choose this path as well the impact upon the other members of the community who do not participate. It must include longitudinal epidemiological and health service studies examining patterns of care for those who participate and those who do not, with a key focus on groups who may be considered at risk of inequity. It must include longitudinal qualitative studies of patients’ views—both those who do and do not seek to access MHD, as well as those of their families in their bereavement. It must include studies of practitioners exploring job satisfaction, burnout, the time commitment of providing care, the impact upon individual practitioners, upon teams and upon systems of care. It must delineate and explore the effectiveness of different approaches to support of professional staff involved in care and in delivery of MHD. Moreover, it must seek to document through both quantitative and qualitative means the attitudes of the public to these practices along with the attitudes of the public institutions such as professional health and legal organisations who seek to serve the public interests.

All threads must be coordinated, systematically and adequately funded from the outset, and must accompany any legislative change, such that the potential success or otherwise of the legislation is understood.

## Conclusion

It is likely that in Australia, as in other settings [14, 46], requests for MHD will increase over time. It is also likely that anxiety about MHD will diminish and tolerance will increase, on the part of health care workers in general, and palliative care workers more specifically. The availability of MHD has enabled a number of people to access a mode of dying that they felt would provide the relief they sought, as well as acknowledging the terms on which they wished to live—and end—their lives.

It is not possible to tell what impact the increased accessibility of MHD will have on those who do not choose to die in this way or on their families, their professional careers, and the community more broadly.

A major outstanding problem is the lack of an agreed, comprehensive approach to evaluation. The establishment of such a standard is urgently required, both for the purposes of research and to assist with the ongoing refinement of legislative approaches.

## Data Availability

The data that support the findings of this study are available from the corresponding author, [JP], upon reasonable request.
