# Correction to: Voluntary Assisted Dying/Euthanasia: Will This Have an Impact on Cancer Care in Future Years?

**DOI:** 10.1007/s11864-023-01133-9

**Published:** 2023-09-18

**Authors:** Jennifer Philip, Brian Le, Camille La Brooy, Ian Olver, Ian Kerridge, Paul Komesaroff

**Affiliations:** 1 Department of Medicine, University of Melbourne, Victoria Pde, Fitzroy, Melbourne, Victoria 3065 Australia; 2 Palliative Care Service, St Vincent’s Hospital, Melbourne, Victoria Australia; 3 Parkville Integrated Palliative Care Service, Peter MacCallum Cancer Centre & Royal Melbourne Hospital, Melbourne, Victoria Australia; 4 Public Health & Preventive Medicine, Monash University, Monash, Victoria Australia; 5 University of Notre Dame of Australia, Sydney, NSW Australia; 6 Haematology Department, Royal North Shore Hospital, St Leonards, NSW Australia; 7 Sydney Health Ethics, University of Sydney, Sydney, NSW Australia; 8 Department of Philosophy, Macquarie University, Macquarie, NSW Australia; 9 Monash University, Monash, Vic Australia

**Correction to: Current Treatment Options in Oncology**

10.1007/s11864-023-01126-8

The original version of this article, unfortunately, contained mistakes. The 2nd author name in the author group was incorrectly written as Camille Le Brooy. The correct spelling should be Camille La Brooy.

The original article has been corrected.

